# Antibody–Drug Conjugates in HR+ Breast Cancer: Where Are We Now and Where Are We Heading?

**DOI:** 10.3390/jcm12237325

**Published:** 2023-11-26

**Authors:** Pierluigi De Santis, Valeria Sanna, Martina Perrone, Chiara Guarini, Anna Natalizia Santoro, Carmelo Laface, Daniela Carrozzo, Gaia Rachele Oliva, Alessandro Fancellu, Palma Fedele

**Affiliations:** 1 Oncology Unit, Francavilla Fontana Ceglie Messapica Hospital District, 72021 Francavilla Fontana, Italy; pierluigi.desantis@asl.brindisi.it (P.D.S.);; 2 Unit of Medical Oncology, A.O.U. Sassari, 07100 Sassari, Italy; valeria.sanna@aouss.it; 3 Faculty of Medicine and Surgery, Università Cattolica del Sacro Cuore, 00168 Roma, Italy; gaiarachele.oliva01@icatt.it; 4 Unit of General Surgery, Department of Medical, Surgical and Experimental Sciences, University of Sassari, 07100 Sassari, Italy; afancel@uniss.it

**Keywords:** HR+ breast cancer, ADCs, target therapy, HER2, combination therapy, trastuzumab deruxtecan, sacituzumab govitecan

## Abstract

Hormone receptor-positive (HR+) breast cancer (BC) accounts for about 60–70% of all diagnosed BCs, and endocrine therapy has long been the hallmark of systemic treatment for this tumor subtype. However, the therapeutic paradigm of luminal BC has been overcome due to recent evidence of antibody–drug conjugate (ADC) activity (such as trastuzumab deruxtecan and sacituzumab govitecan) in pretreated metastatic HR+ BC patients. Therefore, nowadays, the identification of patients who can benefit more from this approach represents a new challenge, as does the management of new toxicities and the integration of these drugs into the therapeutic algorithm of HR+ metastatic BC patients.

## 1. Introduction

Hormone receptor-positive (HR+) human epidermal growth factor receptor 2-negative (HER2−) breast cancer (BC) constitutes nearly 60–70% of all BC diagnoses [1]. This subtype includes more frequent classical tumor histologies (non-special type—NST) and other less frequent ones characterized by different levels of aggressiveness and biological behavior. Invasive lobular carcinoma (ILC) represents the second most common BC histological subtype. It is typically less aggressive, with a strong estrogen and/or progesterone receptor positivity and HER2 negativity [2], tending to be endocrine-responsive [3] and resistant to chemotherapy [4]. Conversely, invasive micropapillary breast cancer (IMBC), a rare histological subtype, is characterized by a marked propensity for lymphovascular invasion [5]. HER2 represents a prognostic and predictive factor for patients with BC. The clinical relevance of HER2 positivity in patients with ductal carcinoma in situ (DCIS) is still unclear [6].

In HR + HER2− BC, the inhibition of the estrogen receptor (ER) pathway through endocrine therapies (ETs) represents the primary therapeutic approach, both in the early and advanced stages [1]. The strong reliance of BC on the ER pathway for progression, survival, and advancement is well-established and estrogen receptor alpha (ERα), encoded by the Estrogen receptor 1 (ESR1) gene, plays an important role [7].

There are both drugs that directly interact with Erα, such as selective estrogen receptor modulators (SERMs), and others that reduce circulating estrogen levels. The most commonly used SERM is tamoxifen, particularly in the adjuvant setting [8]. Tamoxifen restrains the growth of BC cells by competitively antagonizing the ER; however, it causes well-documented estrogen-like adverse events in the genital tract, in the hepatic and bone metabolism, and in the coagulative pattern [9]. Tamoxifen is the preferred ET for adjuvant treatment of premenopausal women with low-risk cancers, and it can also be considered for postmenopausal women who either cannot receive aromatase inhibitors (AIs) or experience poor tolerance to them [10].

A reduction in endogenous estrogen production can also be obtained by deactivating aromatase, the enzyme responsible for converting androgens to estrogens in peripheral tissues.

AIs are the standard of care for postmenopausal women and are frequently employed for treating premenopausal women at high risk of recurrence, often in conjunction with ovarian function suppression [8]. In the metastatic setting, recent combination strategies, particularly those involving inhibitors of cyclin-dependent kinase 4 and 6 (CDK4/6), mammalian target of rapamycin (mTOR), phosphatidylinositol 3-kinase (PI3K), and serine-threonine kinase 1 (AKT), have demonstrated improved survival outcomes when combined with an AI or fulvestrant, compared to ET alone [1,10,11]. Consequently, AIs in combination with CDK4/6 inhibitors are currently approved as a first-line treatment for advanced BC, while the combination of AI plus the mTOR inhibitor, everolimus, has been approved for subsequent lines of therapy [1].

The occurrence of endocrine resistance is often the reason for the progression of disease (PD) during ET [11]. ESR1 gene mutations are a proven driver of ET resistance, mainly due to a structural alteration of ER [12]. ESR1 mutations are mainly found in metastatic disease, occurring in approximately 1/3 of patients treated with AIs, indicating the occurrence of endocrine resistance [13]. Selective estrogen receptor degraders (SERDs) like fulvestrant have shown the ability to overcome endocrine resistance.

**Figure 1:**
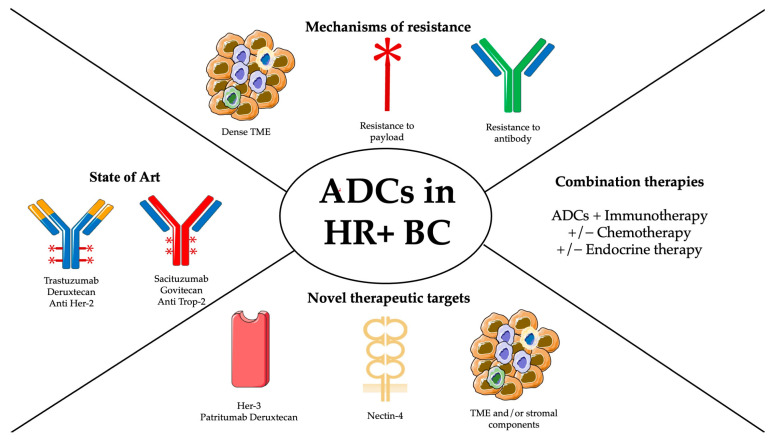
Summary figure.

As a combination treatment, fulvestrant plus CDK4/6 inhibitors have been approved as first- or second-line therapy. Fulvestrant has been also approved in combination with the PI3K inhibitor alpelisib for PIK3CA-mutant HR+, HER2− locally advanced or metastatic BC after progression from first-line treatment with a single agent hormonal therapy [14,15]. More recently, the treatment paradigm for this subgroup has further evolved with the advent of ADCs, which have been shown, due to their mechanism of action, to be capable of providing tumor responses independently of the reference biomarkers detected. Therefore, this review aims to highlight the state of the art in the use of ADCs in HR + BC, focusing on their mechanism of action and resistance mechanisms, and to explain their future potential (Figure 1). 

## 2. Antibody–Drug Conjugates (ADCs): Structure and Mechanism of Action

### 2.1. Structure

ADCs represent a class of targeted anticancer agents that have recently achieved considerable development. They are antibody conjugates that combine the targeting capacity of mAb against selective tumor antigens with the cytotoxic effects of chemotherapeutic agents [16].

ADCs are composed of three components:

(1) A monoclonal humanized antibody (mAb): it can target tumor antigens or proteins expressed mainly in tumors rather than normal cells;
(2) A cytotoxic payload carried by the mAb and released in tumor cells, where it exerts its cytotoxic activity. ADCs in clinical development carry various classes of cytotoxics, including microtubule inhibitors (e.g., auristatins like monomethyl auristatin E and F, MMAE, and MMAF, and maytansinoid microtubule inhibitors, DM1 and DM4), antitumor antibiotics (calicheamicins), and DNA-targeting agents (e.g., camptothecin analogs like SN-38 and exatecan mesylate). ADC payloads are potent cytotoxic agents with IC50 values in the nanomolar to picomolar range, regardless of the specific cytotoxic mechanism;
(3) A linker: this component of the ADC connects the mAb to the cytotoxic payloads. The biological properties of linkers are crucial for safety and antitumor activity, as they should ensure the stability of the entire ADC complex in the bloodstream while enabling optimal intratumoral delivery of the payload for maximum efficacy. Linkers are categorized as cleavable and non-cleavable (stable), according to their capacity to undergo enzymatic or chemical cleavage [17].

### 2.2. Mechanism of Action

ADCs are developed to selectively deliver cytotoxic drugs to tumor cells expressing a specific target antigen, thereby promoting tumor-specific cytotoxicity. This process involves several steps, including:

- Recognition of the target antigen on the tumor cell (Phase 1);
- Internalization of the ADC/antigen complex via endocytosis within the tumor cell (Phase 2);
- Transfer of the cytotoxic payload to the tumor cell’s lysosomes (Phase 3);
- Release of the cytotoxic payload within the tumor cell thanks to its acidic environment and presence of proteolytic enzymes (Phase 4);
- Cytotoxicity mediated by the cytotoxic payload, leading to cell death (Phase 5) [18].

Additionally, ADCs can induce antibody-dependent cellular cytotoxicity (ADCC) through the antibody portion, mediated by natural killer (NK) cells binding to the Fc portion of IgG mAbs via the CD16 receptor.

Although ADCs are designed for the specific killing of tumor cells expressing the target antigen, they can also exert a bystander effect by affecting neighboring tumor cells not expressing the antigen [19]. This phenomenon involves the release of the cytotoxic drug into the tumor microenvironment (TME), allowing it to penetrate the plasma membrane of nearby tumor cells regardless of antigen expression [20]. These mechanisms collectively contribute to the antitumor activity of ADCs, even in cases with heterogeneous levels of target antigen expression.

**Figure 2:**
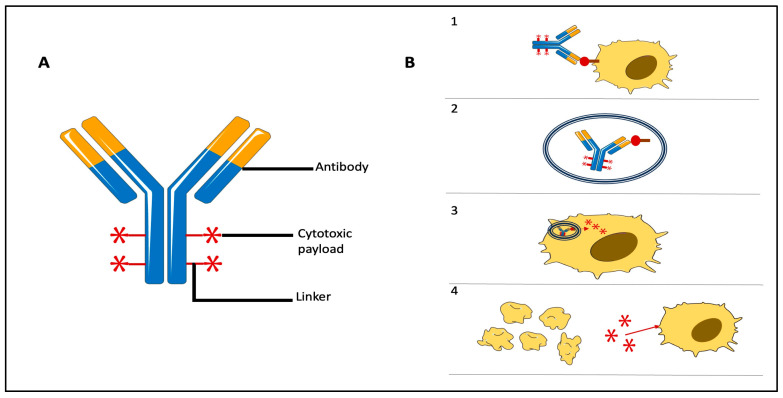
(**A**). Structure of ADCs. (**B**). Mechanisms of action of ADCs: (1) Identification of target and creation of ADC/antigen complex. (2) Intracellular internalization of ADC/antigen complex. (3) Release of cytotoxic payload. (4) Cell death and involvement of other tumor cells thanks to bystander effect.

The mechanisms of action of ADCs are summarized in Figure 2.

### 2.3. Mechanism of Resistance

ADCs have had an important clinical impact on BC patients; however, a percentage of these patients exhibit primary or secondary resistance to ADCs without an initial tumor response. Mechanisms of resistance in ADCs are related to:

- Resistance against the entire ADC complex through physical barriers caused by a dense TME that hinders the intratumoral spread of the ADC [21];
- Resistance against the antibody caused by antigen loss [22] or derangement in internalization [23];
- Resistance against the payload caused by alterations in the payload target [24] or alternative activation of signaling pathways [25].

A better understanding of how the TME modulates responses to ADCs and of the above-mentioned mechanisms of resistance could lead to an improvement of the treatment effects. Upcoming studies on ADCs should be focused on strategies to overcome this resistance and enhance efficacy by recurring to new combination strategies with signaling pathway inhibitors and ICIs, targeting TME components, or discovering new payloads.

### 2.4. Trastuzumab Deruxtecan (T-Dxd)

T-DXd is an ADC with a humanized HER2 antibody (trastuzumab—T) covalently linked to a Topoisomerase I inhibitor (DXd). It includes a potent payload, DXd, a novel linker with a higher drug-to-antibody ratio (DAR) of 8, and a tumor-selective cleavable linker sensitive to lysosomal proteases. Therefore, its shorter systemic half-life permits it to avoid systemic exposure and the bystander effect promotes the spread of cytotoxic effects of Dxd in the TME [26].

In DestinyBreast-01, a phase 2 single-arm study, T-DXd showed high antitumor activity in patients with HER2-positive mBC who had formerly received trastuzumab-emtansine (T-DM1) [27]. Subsequently, T-DXd was approved by the Food and Drug Administration (FDA) following the DestinyBreast-03trial, a phase 3 trial in which T-DXd showed a statistically significative improvement in progression-free survival (PFS) compared to T-DM1 in patients with HER2-positive mBC [28,29], becoming the gold standard in the second line of treatment of HER-2-positive BC.

### 2.5. Enfortumab Vedotin (EV)

EV is an ADC targeting Nectin 4 composed of a fully humanized IgG1 antibody, with MMAE as the cytotoxic payload [30]. In preclinical studies, both in vitro and in vivo, antitumor activity has been demonstrated for both HR-positive and triple-negative breast cancer (TNBC), resulting in the complete eradication of established tumors. This ADC is currently approved for pretreated, locally advanced or metastatic urothelial carcinoma patients, according to the results of the EV-301 study [31].

### 2.6. Sacituzumab Govitecan

Sacituzumab govitecan is an ADC composed of a humanized antitrophoblast cell-surface antigen-2 (TROP-2) monoclonal antibody linked through a pH-sensitive cleavable linker to 7–8 molecules of an active metabolite of irinotecan (SN-38) [32]. Preclinical studies showed an important in vitro and in vivo antitumor activity in animal models with TNBC cell lines. A phase 2 study (IMMU-132-01) evaluated SG as a single agent in heavily pretreated patients (median of previous lines of therapies: five) with metastatic TNBC [33]. Based on these results, sacituzumab obtained accelerated FDA approval for patients with metastatic TNBC who had previously been subjected to at least two prior lines of treatment for metastatic disease [34]. More recently, the ASCENT phase 3 randomized trial confirmed sacituzumab’s efficacy in pretreated patients with metastatic TNBC [35].

### 2.7. Patritumab Deruxtecan

Patritumab deruxtecan (U3-1402) is an ADC targeting HER3 composed of patritumab (U3-1287), a humanized anti-HER3 mAb carrying another metabolite of irinotecan (exatecan) through a peptide linker. It was first evaluated in HER3-expressing tumor cell lines and xenograft models with varying HER3 expressions, demonstrating a specific binding to HER3 and an efficient tumor cell internalization and killing through the release of the cytotoxic payload, leading to DNA damage and, consequently, to tumor cell apoptosis [36].

## 3. ADCs in Metastatic HR+ Breast Cancer: State of the Art

### 3.1. Trastuzumab-Deruxtecan (T-DXd) in HR + BC

A phase Ib study evaluated T-Dxd in 54 patients with mBC exhibiting low HER2 expression. In this heavily pretreated patient cohort, relevant antitumor activity was observed: the objective response rate (ORR), evaluated by an independent central review, was 20/54 (37.0%; 95% CI, 24.3% to 51.3%), with a median response duration of 10.4 months (95% CI). Of interest, there was a numerical difference in terms of ORR based on HR status, specifically 40.4% in HR+ patients and 14.3% in those with TNBC. This outcome underlines the biochemical differences between cases of low HER-2-expressing HR+ and TNBC, as the former appears to have a different transcriptomic profile driven by HER2 expression [37]. Based on these results, a randomized phase 3 trial DESTINY-Breast 04 was initiated, comparing T-Dxd with physician’s choice chemotherapeutic treatments for patients pretreated with 1–2 lines of chemotherapy, including TNBC. The median follow-up was 18.4 months (mos; 95% CI, 17.9–19.1). This represents the first phase 3 study on HER2-targeted therapy in patients with low HER-2 expression, demonstrating statistically and clinically significant benefits in PFS and overall survival (OS) compared to standard chemotherapy. The median treatment duration was 8.2 months (range, 0.2–33.3) with T-DXd and 3.5 months (range, 0.3–17.6) with chemotherapy. Grade (G) ≥ 3 treatment-related adverse events (TEAEs) occurred in 52.6% of T-DXd-treated patients compared to 67.4% of chemotherapy-treated patients [38]. Safety data were published at ESMO Breast Cancer 2023, confirming a manageable safety profile of T-Dxd. Thirteen patients had shown drug-related grade 1 interstitial lung disease (ILD), six of whom had resumed T-DXd after resolution. Incidence of any-grade drug-related neutropenia (NP) and febrile NP was lower for T-DXd vs. chemotherapy; nausea/vomiting (N/V) events in T-DXd vs. chemotherapy were 79.5% vs. 35.5%, but 92.3% of T-DXd and 68.8% of chemotherapy N/V events were resolved by antiemetic prophylaxis. For T-DXd, the incidence of grade ≥ 3 TEAEs and TEAEs associated with drug discontinuations (DDs) was higher in over-65-year-old patients; ILD/pneumonitis was the main cause of DD.

Data were also published regarding the subgroup of patients with low ER expression (1–10%). Patients treated with T-DXd had better PFS (mPFS, 8.4 months vs. 2.6 months; HR 0.24 [95% CI, 0.12–0.48]) and OS (HR 0.35 [95% CI, 0.16–0.75]) vs. the chemotherapy group, with a safety profile consistent with primary analyses [39].

Therefore, a recent phase 2 trial evaluated T-DXd in patients with pretreated mBC according to different HER2 levels of expression (71.5% of patients were hormone receptor-positive). The study confirmed that HER2 status is a predictive biomarker of responsivity to T-DXd; however, an important activity of T-Dxd was also confirmed in patients whose cancer expressed lower levels of HER2 or did not express HER2. This implies different mechanisms affecting T-Dxd efficacy and different resistance mechanisms independent from HER2. Further, the trial suggests that the current method of analysis of HER2, immunohistochemistry (IHC), might not be sufficient to define the threshold of HER2 expression and to predict efficacy in patients with HER2-low mBC [40].

### 3.2. Sacituzumab Govitecan (SG) in HR+ Breast Cancer

SG has shown encouraging activity in previously treated HR + HER2-negative mBC in a phase 1/2, single-arm, basket trial. TROP-2 is expressed in epithelial cancers, including HR + mBC, and it is associated with worse survival. Based on these data, the randomized phase 3 study TROPICS-02 (NCT03901339) was planned. In this trial, 543 patients were randomized 1:1 to receive sacituzumab govitecan or TPC. Patients had to have been pretreated with at least two prior chemotherapies for metastatic disease (one of which could be in the neoadjuvant or adjuvant setting if BC recurrence occurred within 12 months). TPC could be eribulin (n = 130), vinorelbine (n = 63), gemcitabine (n = 56), or capecitabine (n = 22). Stratification was performed by the number of prior chemotherapy regimens for the metastatic setting, presence of visceral metastasis, and the use of endocrine therapy in metastatic disease for at least 6 months. The primary endpoint was PFS. Median PFS was 5.5 months (95% CI: 4.2, 7.0) in the SG arm and 4 months (95% CI: 3.1, 4.4) in the monotherapy TPC arm (hazard ratio [HR] of 0.661 [95% CI: 0.529, 0.826]; *p*-value = 0.0003) [41]. The secondary efficacy outcome measure was OS. Median OS was 14.4 months in SG arm (95% CI: 13.0, 15.7) and 11.2 months (95% CI: 10.1, 12.7) in TPC group (HR of 0.789 [95% CI: 0.646, 0.964]; *p*-value = 0.02), resulting in a 21% relative reduction in the risk of death across all subgroups and regardless of TROP-2 status. The most common AEs (≥25%) in the SG arm were a decreased leukocyte and neutrophil count (88% and 83%), anemia (73%), leukopenia (65%), diarrhea (62%), fatigue (60%), nausea (59%), alopecia (48%), hyperglycemia (37%), constipation (34%), and hypoalbuminemia (32%) [42]. A post hoc safety analysis was performed by UGT1A1 status that confirmed a manageable safety profile consistent with previous results of the ASCENT trial, regardless of UGT1A1 status [43]. SG was approved by the FDA in February 2023 with the following indication: unresectable locally advanced HR-positive, HER2-negative (IHC 0, IHC 1+ or IHC 2+/ISH−) BC patients who received endocrine-based therapy and at least other two systemic treatments for metastatic disease [44].

## 4. Future Perspectives

Recent advances in technology have led to an evolution of ADCs with reduced toxicity and better efficacy.

We may be capable of creating the most specific antibody, linker, and payload based on a patient’s tumor antigens and other intrinsic characteristics of the tumor cells and of the tumor microenvironment. The challenge is generating personalized ADCs without prohibitively high costs and within an acceptable timeframe.

Here, we report data from the most advanced studies and a selection of data on the most promising molecules in the setting of HR+ early and metastatic BC (Table 1).

### 4.1. Agents That Target HER2

The TRIO-US B-12 TALENT (NCT04553770) phase 2 trial was designed to assess the clinical efficacy and safety of neoadjuvant T-DXd as a single agent or in combination with ET in HR+/HER2-low early BC. Patients with tumors larger than 2 cm are included. Patients are scheduled to receive six to eight cycles of T-DXd (5.4 mg/kg IV q21 days) alone or in combination with anastrozole (1 mg PO QD). Pre- and perimenopausal patients with BC randomized to the AI arm also take GnRH agonists. The primary objective is to identify the treatment arm with better efficacy, based on the pathological complete response (pCR) rate. Other endpoints include treatment safety, changes in Ki-67 expression, biomarker analysis (including serial cfDNA analysis), residual cancer burden (RCB) index, and health-related quality of life (HrQOL). Study completion is scheduled for September 2025. As of October 2022, 58 participants have been enrolled, and 33 have completed treatment [45].

Up to date, no trials on the use of T-DXd in HER2-low HR+ in the adjuvant setting are available.

The DESTINY-Breast06 (NCT04494425) phase 3 study evaluates T-DXd versus TPC (capecitabine, paclitaxel, or nab-paclitaxel) in patients with HR+, HER-2-low-expressing BC with PD during ET. Prior chemotherapy for mBC is considered an exclusion criterion. The primary objective of the study is PFS among HR+, HER-2-low BC patients. The enrollment end date was December 2022, with an enrollment goal of 866 patients. Study completion is scheduled for July 2026. No interim results are available [46].

SYD985, also known as vic-trastuzumab duocarmazine, is an ADC with a humanized anti-HER2 antibody covalently linked to a duocarmazine prodrug [47,48] which, in a phase 1 study, demonstrated notable clinical efficacy in extensively treated BC patients with different expressions of HER2, leading to an accelerated FDA approval. Furthermore, in subsequent studies, SYD985 demonstrated positive outcomes compared to standard treatments in the phase 3 TULIP study. In this study, SYD985 reached its primary endpoint, improving mPFS (7.0 vs. 4.9 months) and tending towards an OS advantage, showing a good tolerability profile.

Disitamab vedotin (RC48) comprises a mAb targeting HER2, hertuzumab, accompanied by a MMAE payload. Hertuzumab demonstrated an enhanced affinity for HER2 and an increased capacity to trigger immune-mediated cytotoxicity [49]. In the escalating-dose C001 CANCER phase 1 trial, RC48 demonstrated effectiveness regardless of HER2 expression. Within the HER2-low expression subpopulation, the ORR was 39.6%, while PFS was 39.6%, respectively, with an RC48 dose of 2 mg/kg [50].

### 4.2. Agents That Target HER 3

Patritumab deruxtecan (U31402-A-J101—P-Dxd) is a novel ADC consisting of a human anti-HER3 monoclonal antibody covalently linked to the topoisomerase I payload (HER3-DXd). In the SOLTI-1805 trial, TOT-HER3 was investigated for the potential biological effect of P-DxD in HR+/Her2-negative BC, showing that ERB3 gene expression is predominant in this subgroup, suggesting a role for anti-HER3 therapies in HR+/HER-2-negative BC [51].

P-DxD was studied in this phase 1/2, multicenter, open-label trial (NCT02980341) in patients with previously treated metastatic BC. The primary endpoint of this trial was the assessment of safety and efficacy; secondary endpoints included the assessment of the relationship between efficacy and HER3 expression. Patients had a median of five previous systemic treatments for locally advanced/metastatic disease and a performance status ECOG of 0–1. The first results highlighted that U3-1402 shows antitumor activity in pretreated HER3-expressing mBC patients, and treatment is associated with a manageable safety profile. The median treatment duration with HER3-DXd was 5.9 months (range, 0.7–30.6). Efficacy was shown in patients with HR+/HER-2-negative metastatic BC, TNBC, and HER-2+ metastatic BC. The ORR was 33% and the disease control rate (DCR = CR + PR + SD) was 95%. A total of 71.4% of the 130 patients had grade ≥ 3 TEAEs, of which the most common were decreased neutrophil count (39.6%), thrombocytopenia (30.8%), and anemia (18.7%) [52].

Up to date, no trials on the use of P-DXd in the adjuvant or neoadjuvant setting are available.

### 4.3. Agents That Target TROP-2

Up to date, no trials on the use of SG in the adjuvant or neoadjuvant setting are available.

Datopotamab deruxtecan (Dato-DXd) represents a third generation of ADCs designed to target TROP-2. This ADC consists of an IgG1 monoclonal antibody linked to a topoisomerase-I inhibitor (MAAA-1181a, a derivative of exatecan) via a tetrapeptide-based cleavable linker, maintaining a DAR of 4:1 [53]. Numerous phase 1–3 trials are currently in progress to evaluate the efficacy of this new ADC, either as a monotherapy or in combination with ICIs. In the TROPI-ON-PanTumor01 study (NCT03401385), in a cohort of patients previously treated for HR+/HER2- mBC, an ORR of 29% and a DCR of 85% were observed. Predominant AEs included stomatitis (80%), nausea (56%), fatigue (46%), and alopecia (37%) [54].

### 4.4. Novel Selected Targets

LIV-1 is a zinc transporter that can be found in various tissues. Approximately 90% of both HR + BC and TNBC cases exhibit moderate-to-high LIV-1 expression [55] and they are characterized by a more aggressive behavior in terms of disease dissemination [56].

Ladiratuzumab vedotin (SGN-LIV1) is an ADC which targets LIV-1, conjugated to a MMAE payload, analogous of dolastatin 10, and capable of inducing cell arrest proliferation, consequently leading to cell apoptosis. An ongoing phase 1 dose escalation and expansion trial is currently enrolling patients with metastatic LIV-1-overexpressed BC (TNBC or endocrine-resistant HR+) that have received at least two prior cytotoxic regimens (NCT01969643). Preclinical evidence suggests that SGN-LIV1 can promote the release of immunogenic cell death (ICD) [57].

Nectin-4 is an adhesion molecule involved in cell proliferation and migration. This protein is present in approximately two-thirds of urothelial carcinomas and in BCs, while it is less expressed in normal tissues [58]. EV represents a novel ADC which targets nectin-4, comprising a fully humanized IgG1 antibody linked to a MMAE payload. Currently, a multicohort, phase 2 clinical trial (NCT04225117) is evaluating EV’s efficacy and safety in patients with different advanced solid tumors, including those with heavily pretreated HR + BC and TNBC [59].

Currently, ADCs require internalization to be able to release the cytotoxic payload and this issue represents a therapeutic hurdle. A possible alternative strategy is the extracellular releasing of the payload binding itself to other components of a tumor cell, such as target cell-surface antigens, antigens, and secreted proteins in the TME or stromal and vasculature components. This may remediate the dependence on the overexpression of antigens, overcoming a possible ineffective internalization, and can potentially increase the number and the types of cancer targets in the extracellular tumor matrix, maximizing therapeutic effects [60].

### 4.5. ADCs in Combination

There is a strong rationale for combining ADCs and immunotherapy. Therefore, ADCs can induce cell surface presentation in dying cells, leading to the exposition of damage-associated molecular patterns (DAMPs) that are highly immunogenic. Moreover, the effector immune system maintains its functions of activating ADCC. We also know that microtubule-inhibitor payloads such as maytansinoids, dolastatins, auristatins, and topoisomerase-I inhibitor payloads can induce dendritic cell activation directly [61].

Different combination strategies in BC are currently being investigated in phase 1–2 trials.

Destiny-Breast08, a multicenter, open-label, two-part trial evaluating five T-DXd combinations in HER2-low mBC patients is ongoing. The combinations in HR + mBC are with capecitabine, capivasertib, durvalumab, paclitaxel, anastrozole, and fulvestrant. The first results underline that T-DXd can be employed safely in combination with AIs (anastrozole) or SERDs (fulvestrant); however, additional results are expected in the next few months (NCT04556773). Finally, the Morpheus-panBC trial, an umbrella study, is evaluating multiple combinations of chemotherapy, immunotherapy, ADCs, and hormonal therapy in locally advanced TN or HR+ mBC [62,63,64,65].

## 5. Conclusions

The landscape of HR + BC treatment has significantly evolved with the introduction of ADCs, marking a new era in targeted therapy. HR + BC, accounting for a substantial portion of diagnosed cases, has long been managed through ET. However, the challenge of de novo and acquired resistance has pushed researchers to explore novel approaches to overcome therapeutic hurdles. ADCs are a unique category of anticancer agents that combine the specificity of monoclonal antibodies with the cytotoxic effects of chemotherapeutic payloads. Structurally, ADCs consist of a monoclonal antibody targeting tumor-associated antigens, a cytotoxic payload, and a linker that connects the two components. The mechanism of action involves target antigen recognition, internalization of the ADC-antigen complex, release of the payload within tumor cells, and subsequent cytotoxicity induction. ADCs also hold the potential for bystander effects, where cytotoxic drugs released by tumor cells can impact neighboring non-targeted cells. T-DXd, a novel ADC targeting HER-2, has demonstrated promising results in HR + BC patients with low HER2 expression. Studies have indicated its significant antitumor activity, with an ORR and median response duration reflecting its efficacy. Notably, T-DXd has shown distinct efficacy based on HR status, suggesting underlying biological differences between the HR+ and TN subtypes. SG, another ADC targeting TROP-2, has shown encouraging activity in HR + HER-2 mBC patients. The TROPICS-02 trial demonstrated improved PFS and OS compared to single-agent chemotherapy. SG received FDA approval for unresectable locally advanced HR+, HER-2-negative BC, providing a new treatment option for heavily pretreated patients. Patritumab deruxtecan (P-Dxd), an ADC targeting HER-3, has exhibited antitumor activity in HR+/HER2-mBC patients. Further investigation is ongoing to define its role in different breast cancer subtypes.

The evolution of ADCs also involves combinations with other therapeutic approaches, particularly immunotherapy. ADCs induce damage-associated molecular patterns (DAMPs) upon cell death, potentially enhancing immune recognition and activation. Combination trials involving ADCs and immune checkpoint inhibitors are being explored, aiming to maximize therapeutic responses and exploit both pathways for enhanced outcomes.

As the landscape continues to evolve, it is becoming evident that personalized approaches are crucial for optimizing ADC therapy. Identifying the right patients, understanding the mechanisms of response and resistance, and managing potential toxicities are all challenges that need to be addressed. Ongoing research and clinical trials will be pivotal in refining the use of ADCs in the treatment of HR + BC. With these advancements, there is hope for improved outcomes and enhanced quality of life for patients facing this challenging cancer subtype.

## Figures and Tables

**Table 1:** Ongoing clinical trials evaluating new antibody–drug conjugates in breast cancer monotherapy in HR + BC.

Antibody-Drug Conjugate	Payload	Linker	Trial	Population	Phase	Status
HER 2						
Vic-trastuzumab duocarmazine (SYD985)	duocarmycin (DNA alkylator)	cleavable	NCT04602117	HER2-positive advanced solid tumors or HER2-low breast cancer	1	recruiting
MRG002	MMAE	cleavable vc-linker	NCT05263869	HER2-positive BC with liver metastases	2	recruiting
			NCT04924699	HER2-positive unresectable locally advanced or metastatic BC	2	recruiting
			NCT04742153	HER2-low, locally advanced or mBC	2	recruiting
Disitamab-vedotin (RC48)	MMAE	cleavable	NCT04400695	locally advanced or HER2-low mBC	3	recruiting
			NCT03052634	advanced BC with HER2-positive or low HER2 expression	1–3	active, not recruiting
HER3						
Antibody–drug conjugate	payload	linker	trial	population	Phase	Status
Patritumab deruxtecan	exatecan derivative	tetrapeptide-based	NCT04965766	first-line HER3-high, HER2−, HR+ unresectable locally advanced BC or mBC	2	recruiting
			NCT04610528	HR+/HER2− tumor with non-metastatic primary invasive BC, untreated and recently diagnosed	1	recruiting
TROP2						
Antibody–drug conjugate	payload	linker	trial	population	Phase	Status
Datopotamab deruxtecan	deruxtecan	tetrapeptide-based cleavable linker	NCT05104866 (TROPION-Breast01)	inoperable or metastatic HR+/HER2 − BC	3	recruiting
Nectin-4						
Antibody–drug conjugate	payload	linker	trial	population	Phase	Status
Enfortumab vedotin	MMAE	protease-cleavable	NCT04225117	treatment of refractory advanced solid tumors (HR+/HER2− or TNBC)	2	active, not recruiting
FRα						
Antibody–drug conjugate	payload	linker	trial	population	Phase	Status
PRO1184	exatecan, a topoisomerase 1 inhibitor	cleavable	NCT05579366	advanced and/or metastatic solid tumors (TNBC, HR+, and HER2+)	1–2	recruiting

## Data Availability

Not applicable.
